# Tumor-associated microglia and macrophages in glioblastoma: From basic insights to therapeutic opportunities

**DOI:** 10.3389/fimmu.2022.964898

**Published:** 2022-07-27

**Authors:** Guoqing Wang, Kunhong Zhong, Zeng Wang, Zongliang Zhang, Xin Tang, Aiping Tong, Liangxue Zhou

**Affiliations:** ^1^ Department of Neurosurgery, West China Hospital, West China Medical School, Sichuan University, Chengdu, China; ^2^ State Key Laboratory of Biotherapy and Cancer Center/Collaborative Innovation Center for Biotherapy, West China Hospital, Sichuan University, Chengdu, China

**Keywords:** glioblastoma, glioma, macrophages, microglia, tumor-associated microglia/macrophages (TAMs), immunotherapy

## Abstract

Glioblastoma (GBM) is the most common and malignant primary brain tumor in adults. Currently, the standard treatment of glioblastoma includes surgery, radiotherapy, and chemotherapy. Despite aggressive treatment, the median survival is only 15 months. GBM progression and therapeutic resistance are the results of the complex interactions between tumor cells and tumor microenvironment (TME). TME consists of several different cell types, such as stromal cells, endothelial cells and immune cells. Although GBM has the immunologically “cold” characteristic with very little lymphocyte infiltration, the TME of GBM can contain more than 30% of tumor-associated microglia and macrophages (TAMs). TAMs can release cytokines and growth factors to promote tumor proliferation, survival and metastasis progression as well as inhibit the function of immune cells. Thus, TAMs are logical therapeutic targets for GBM. In this review, we discussed the characteristics and functions of the TAMs and evaluated the state of the art of TAMs-targeting strategies in GBM. This review helps to understand how TAMs promote GBM progression and summarizes the present therapeutic interventions to target TAMs. It will possibly pave the way for new immune therapeutic avenues for GBM patients.

## Introduction

Glioblastoma (GBM) is the most common and aggressive primary malignant brain tumor in adults with a dismal prognosis and poor quality of life (1). Despite aggressive treatment including surgical resection, targeted radiotherapy and high-dose chemotherapy, GBM patients still only have a median overall survival of 15 months and a 5-year survival rate of less than 3% (2). Some ongoing studies are evaluating the effects of immunotherapy for GBM, despite dramatic responses in some cases, the prognosis of GBM patients remains unfavorable (3). The immunologically “cold” tumor microenvironment (TME) has recently emerged as one of the crucial roles in GBM progression and therapeutic resistance (4). Thus, it may be an optimal strategy to disrupt the barrier of immunosuppression by targeting the genetically stable tumor stroma within the GBM TME rather than the constantly mutating tumor cells (4).

GBM is characterized by a lack of T cell infiltration but robust tumor-associated microglia and macrophages (TAMs) infiltration, which constitute more than 30% of infiltration cells in GBM (5). In addition, TAMs infiltration is associated with GBM progression. Compared with Foxp3^+^ regulatory T (Treg) cells, TAMs are a strong predictor of survival for patients with GBM (6). Mechanistically, TAMs could secret growth factors, cytokines and chemokines to build up and remodel the GBM TME, which enables the tumor cells to proliferate, survive and metastasize (7–9). Accordingly, targeting these tumor-supportive TAMs represents a novel promising treatment strategy to improve the prognosis of GBM patients (10).

In this review, we will discuss the current understanding of the origin, characteristics and functions of the TAMs and evaluate the state of the art of TAM-targeting strategies in GBM. This can help us understand how TAMs affect tumor progression and pave the way to enhance the efficacy of current therapies by targeting or harnessing TAMs for GBM patients.

## Tumor-associated microglia and macrophages in glioblastoma

### Origin of microglia and macrophages

**Figure 1:**
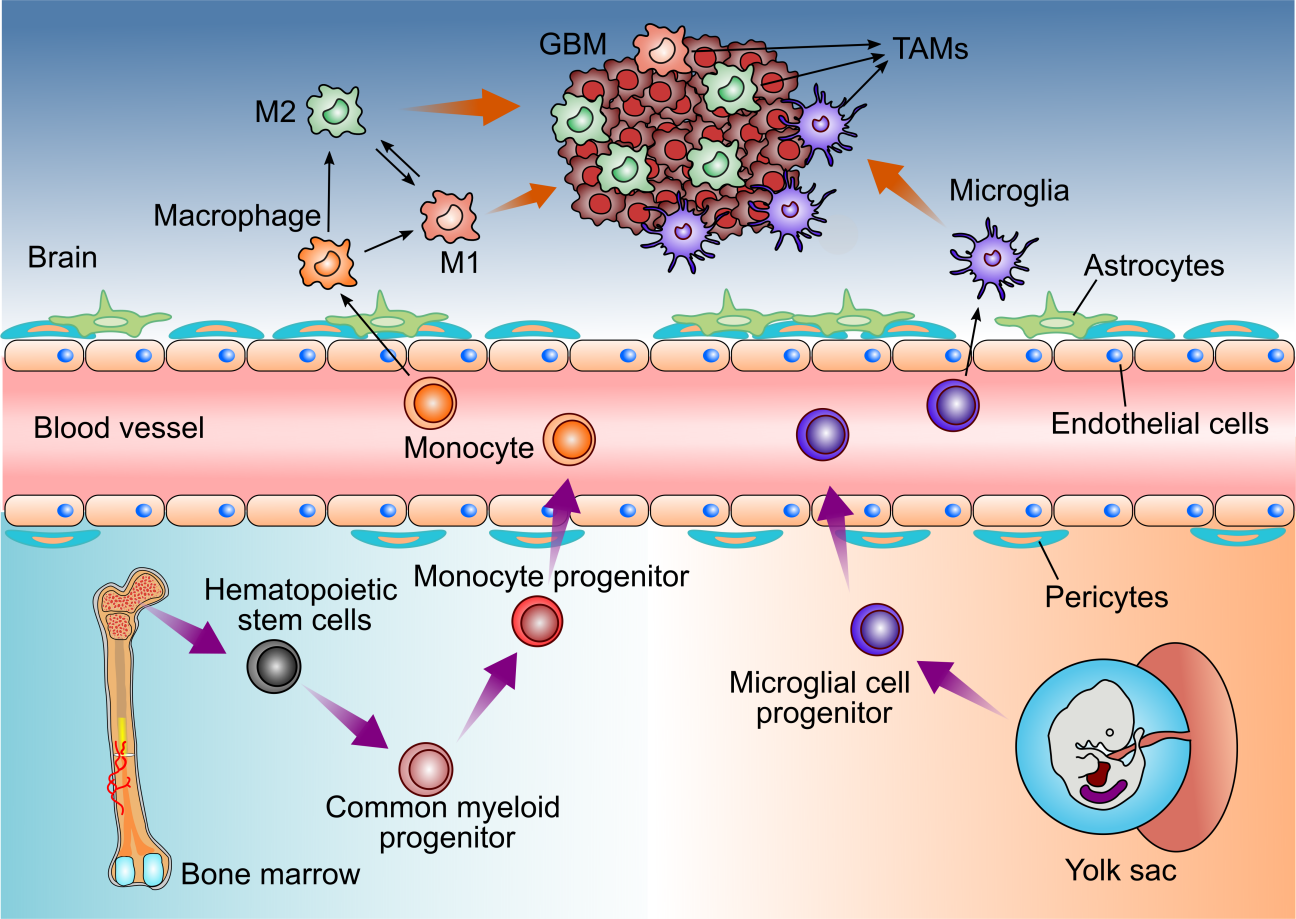
Distinct origins of Glioblastoma-associated Microglia and macrophages. Microglia derive from progenitors of the embryonic yolk sac, while peripheral macrophages are the monocytes deriving from the hematopoietic stem cells in bone marrow. Both cell populations enter the CNS and could be recruited by GBM cells, then referred to as tumor-associated microglia and macrophages (TAMs).

In GBM, TAMs consist of resident microglia and peripheral macrophages recruiting to the tumor tissue (11). Both cell populations have the similar functions in the TME, so they are recognized as one cell cluster (12, 13). However, some studies have found that microglia and macrophages are different with various functions and different origins in GBM (**Figure 1**) (14, 15).

Brain-resident microglia are located in the brain parenchyma of the central nervous system and are derived from the primitive myeloid progenitors (15, 16). It is demonstrated that microglia derive from progenitors of the yolk sac and enter the CNS before embryonic day 8 in mice (15, 17). They are a long-living cell population and maintain the self-renewal capability without contribution from bone marrow-derived progenitors. Thus, microglia are identified as an ontogenically distinct population from the peripheral macrophage (18–21).

In healthy states, brain-resident microglia continuously scan their surroundings in order to maintain brain tissue homeostasis and immune defenses. However, microglia also have a pro-tumor activity in GBM patients (22).

In addition, TAMs also contain peripheral macrophages driven by inflammatory factors from GBM (23). Under the pathological stimulus of GBM, circulating bone marrow monocytes derived from hematopoietic stem cells can migrate to tumor tissue, where they differentiate into monocyte-derived macrophages and promote tumor progression (24, 25).

### Polarization of TAMs

In a simplified model, TAMs are classified as M1 or M2 polarized cells that are relative with pro-inflammatory/anti-tumor or anti-inflammatory/pro-tumor property respectively. Interestingly, these two polarized states can convert from each other (26). Despite being oversimplified, this classification helps us to understand the polarization of TAMs.

This classification is based on some *in vitro* studies which observed the response to inflammations (27–31). However, the situation *in vivo* is more complex and intermediate phenotypes have been identified that do not belong to M1 or M2 polarized cells, suggesting that the transcriptional program of TAMs can be recognized as a dynamic spectrum. For example, different forms such as M2a, M2b and M2c have been proposed to describe the continuous phenotype of M2 polarized cells (32).

In terms of great diversity and plasticity of TAMs, they can acquire various phenotypes in different TME. It is reported that TAMs behave the M1 phenotype and exert anti-tumor activities in the early stage of tumor development (9, 33). The polarization of TAMs from M1 to M2 phenotype is associated with the tumor progression. Generally, TAMs mainly play a role in the initiation, malignant progression, angiogenesis and resistance to treatment in GBM. Thus, in this review, we focus on the pro-tumor effects of TAMs in GBM (5, 34–41).

## Tumor-promoting function of tumor-associated microglia and macrophages in glioblastom*a*

### TAMs promote glioma cells proliferation and invasion

**Figure 2:**
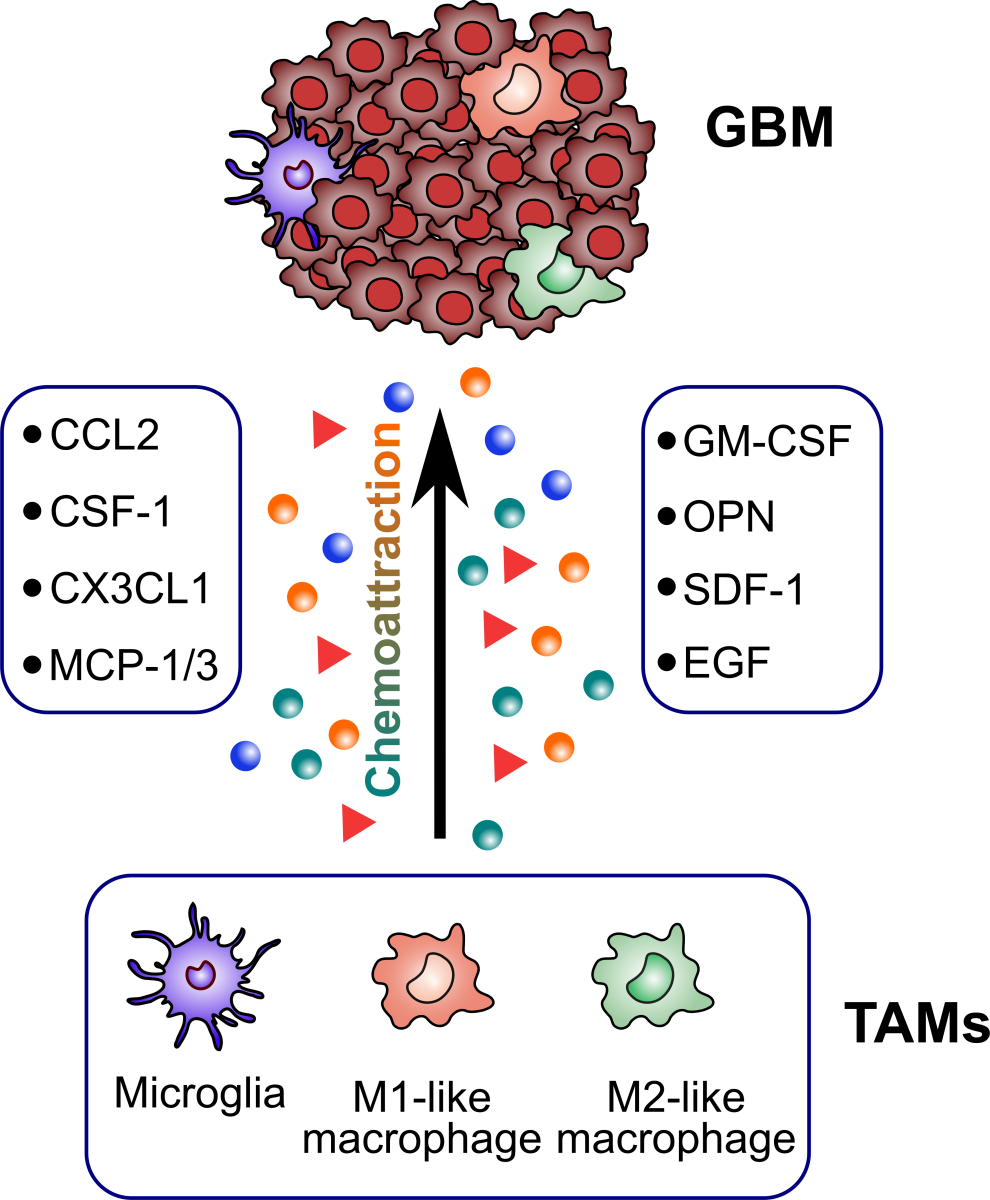
Glioblastoma-derived factors attracting TAMs. TAMs are recruited to the tumor sites by several glioblastoma-derived factors (CCL2, CSF-1, CX3CL1, MCP-1/3, GM-CSF, OPN, SDF-1, EGF).

**Figure 3:**
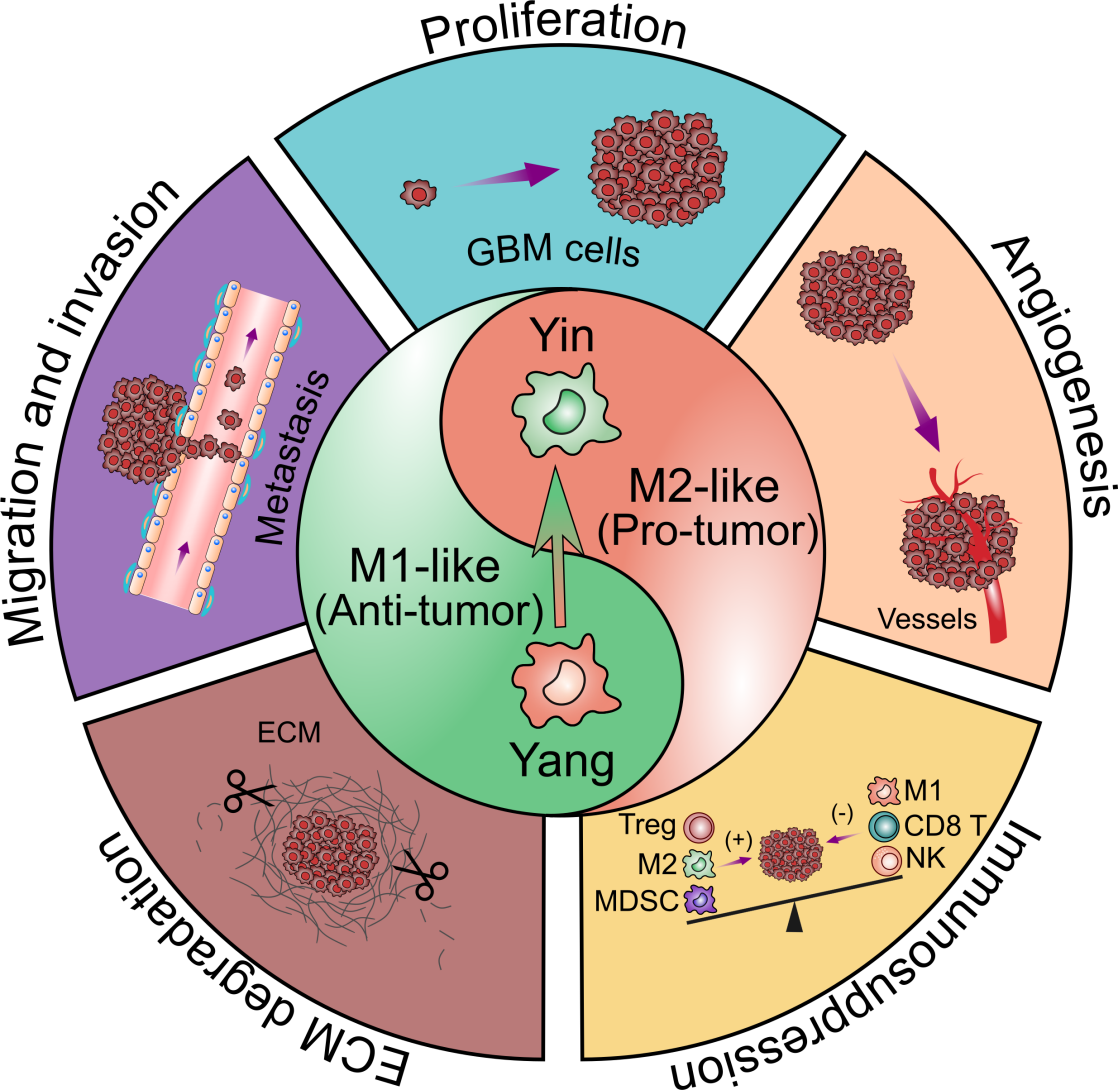
Contribution of glioblastoma-associated microglia and macrophages to tumor progression. TAMs have bimodal, yin and yang effects on immune responses. The yin and yang microglia/macrophage subtypes refer to M2- and M1-like respectively. M1-like TAMs are relative with the pro-inflammatory property while M2-like TAMs are relative with the anti-inflammatory property. The polarization of TAMs from M1 to M2 phenotype is associated with the tumor progression. TAMs are believed to promote GBM progression by several pro-tumorigenic activities including: 1) promoting GBM cells proliferation; 2) promoting GBM cells migration and invasion; 3) promoting angiogenesis in GBM; 4) facilitating extracellular matrix (ECM) degradation; 5) facilitating the immunosuppressive TME.

TAMs constitute up to 30–40% of the bulk tumor mass and predominate the lymphocyte infiltration in GBM, making them an important consideration for their role in GBM initiation and progression. Several factors released by glioma cells can attract TAMs to the tumor sites (**Figure 2**). Then, TAMs could exert multiple pro-tumorigenic activities (**Figure 3**). To date, accumulating studies have demonstrated that factors released by TAMs play an important role in proliferation and invasion of GBM (42).

Microglia and microglia-conditioned medium promote the invasion of glioma *in vitro*, suggesting that substances released by TAMs mediated this effect. In contrast, oligodendroglia and endothelial cells only weakly stimulated glioma cell motility (43). However, this motility-promoting activity was decreased in glioma cells when the transforming growth factor (TGF)-β was knocked down, indicating the invasion of glioma cells is dependent on microglia-derived TGF-β (44). GBM invasion promoted by TGF-β is involved with the upregulation of integrin. TGF-β induces matrix metalloprotein (MMP)-2 expression and suppresses tissue inhibitor of metalloproteinases (TIMP)-2 expression, both of which accelerate the extracellular matrix (ECM) breakdown (45). MMP can not only mediate the degradation of ECM to facilitate the invasion of GBM into the brain parenchyma, but also facilitate the proliferation of GBM. In response to GBM released factors, membrane type 1 metalloprotease (MT1-MMP) is upregulated in TAMs, but not in tumor cells. Interestingly, microglia release TGF-β, which triggers the release of pro-MMP2 from GBM. Pro-MMP2 is then transformed into active MMP2 by TAMs-expressed MT1-MMP. After deletion of MyD88 or p38, the toll-like receptor (TLR) adapter protein, MT1-MMP expression and GBM proliferation is inhibited. Thus, the high expression of MT1-MMP and subsequently increased proliferation capacity is mediated by TAMs’ TLR and the p38 MAPK pathway (46, 47). Canonical NF-κB signaling has an anti-inflammatory role and is required for GBM proliferation (48).

Pleiotrophin (PTN)-PTPRZ1 paracrine signaling can support GBM malignant proliferation. TAMs secrete abundant PTN which binds to its receptor PTPRZ1 to stimulate GBM proliferation (49). In response to GBM secreted factors, TAMs can express high levels of ATX and LPA1 to support GBM proliferation and invasion (50). Co-chaperone stress-inducible protein 1 (STI1) secreted by TAMs promotes proliferation and invasion of GBM *in vitro*. *In vivo*, the STI1 expression is also measured in a GBM model. Interestingly, high expression of STI1 was observed in TAMs but not in peripheral blood monocytes and lymphocytes, suggesting that TAMs-derived STI1 is also modulated by the TME of GBM (51).

The CCL2/CCR2/IL-6 loop also has a role in promoting GBM invasion. Glioma-derived CCL2 acts on microglia and then triggers the production of IL-6 from microglia, which in turn promotes GBM invasion (52). TAMs highly express CCL8 which promotes the pseudopodia formation of GBM cells. CCL8 in the TME can bind to CCR1 and CCR5 on GBM cells and activate ERK1/2 phosphorylation, finally increasing the invasion of GBM (53). In addition, colony-stimulating factor-1 (CSF-1) secreted by GBM is a chemoattractant for TAMs and facilitates its M2-like activation in autochthonous models, while also demonstrating that CSF-1 overexpression induces GBM proliferation (54, 55). Furthermore, CSF-1 and epidermal growth factor (EGF) released by microglia also stimulate GBM invasion (56). Since both microglia and GBM express EGFR, EGF may serve as a paracrine factor to recruit TAMs to the tumor sites, and at the same time, it binds to EGFR of GBM to stimulate GBM invasion (57, 58).

### TAMs promote angiogenesis

Several studies have shown that GBM is characterized by abnormal angiogenesis (59–61). GBM resistance to anti-VEGF therapy is associated with the infiltration of macrophages (62). In GBM, there is an “inflammation-driven angiogenesis” mechanism. M2-like immunosuppressive macrophages promote angiogenesis, while M1-like pro-inflammatory macrophages suppress angiogenesis (63).

Depleting the TAMs *in vivo* reduces the GBM vessel density, suggesting that TAMs are of importance in tumor angiogenesis (64). Interestingly, selective depletion of resident microglia reduced tumoral vessels compared to ablation of the whole TAMs, indicating that resident microglia rather than peripheral macrophages are the crucial modulator to promote the angiogenesis of GBM (64).

TAMs isolated from GL261 glioma overexpress proangiogenic factors such as VEGF and CXCL2. VEGF is a well-known regulator of angiogenesis while CXCL2, a poorly described chemokine, displayed stronger angiogenic activity than VEGF *in vitro* (64). Interaction of the receptor for advanced glycation end products (RAGE) with its ligands can promote tumor angiogenesis. In GBM, the RAGE signaling in TAMs drives angiogenesis (65), during which the activation of RAGE can upregulate the IL-6 expression (66). Bevacizumab, a VEGF-targeting antibody, failed in a clinical trial evaluating the effect of the combination of bevacizumab to radiotherapy-temozolomide (TMZ) for the treatment of newly diagnosed GBM. Besides, the rate of adverse events was higher with bevacizumab than with placebo (67, 68). GBM-derived macrophage colony-stimulating factor (M-CSF) increases the IGFBP1 expression of microglial cells, which is an important effector to promote angiogenesis. Thus, IGFBP1 could be a potential alternate candidate for developing a targeted therapy for GBM (69).

### TAMs promote the immunosuppressive TME

GBM is characterized by the strong immunosuppressive TME. TAMs are important drivers of the local immunosuppressive TME and are relative to GBM progression and resistance to immunomodulating therapeutic strategies (70). M2-like TAMs contribute to the immunosuppressive TME by secreting immunosuppressive factors such as IL-6, TGF-β, IL-10 in GBM, while only low levels of M1-like pro-inflammatory cytokines such as IL-2, IL-12, TNF-α and IFN-γ are detected (52, 71, 72). This TAMs phenotype is modulated by GBM cells or their soluble factors *in vitro*, which in turn promotes the suppressive TME and contributes to immunoevasion of GBM (73).

The immune functions of TAMs isolated from GBM patients were analyzed. The results indicated that the expression of surface major histocompatibility complex class II (MHC class II) and costimulatory molecules CD40, CD80, and CD86 were reduced in TAMs, thus inhibiting antigen cross-presentation and T cells activation (74, 75). Similarly, in a rodent glioma model, the MHC II and B7 costimulatory molecules were significantly reduced when compared with the normal brain (76). mTOR-dependent regulation of STAT3 and NF-κB activity mediated the immunosuppressive function of TAMs. It plays a role in GBM immune evasion by suppressing the effector T cells infiltration, proliferation and immune function (77). In both *in vitro* and *in vivo* GBM mouse models, the activation of mTOR signaling was observed in the microglia but not in bone marrow-derived macrophages. The activation of mTOR signaling increased phosphorylation of STAT3 and inhibited the NF-κB pathway in microglia, thereby upregulating the expression of IL6 and IL10 (the anti-inflammatory M2-like cytokines) with a concomitant reduction in expression of IL12 (the pro-inflammatory M1-like cytokines) (77). The upregulation of STAT3 in TAMs of GBM has been previously reported (78, 79). It is associated with higher GBM grade and expression of anti-inflammatory cytokines (79). NF-κB is downregulated in TAMs, leading to reduced expression of inflammatory TLR, resulting in the impairment of anti-tumor immune responses in GBM (80). However, although another study found substantial expression of TLRs in TAMs, they were not stimulated to produce pro-inflammatory cytokines (75).

FasL/Fas pathway plays an important role in immunosuppressive TME in GBM (81). T cells invading GBM express Fas receptor, which can directly contact with FasL-expressing GBM cells, and thus induce the apoptosis of T cells (82). Immunoblotting indicated that the membrane-bound FasL expressed nearly twice as much when murine G26 gliomas were implanted intracranially as compared to subcutaneously. Interestingly, microglia were absent in the subcutaneous tumors. Microglia are a major source of FasL expression in GBM and possibly contribute to the local immunosuppressive TME of GBM, which was mediated by the apoptosis of T cells *via* FasL/Fas interaction (81, 83). However, in TAMs isolated from GBM patients, the expression of FasL was low to absent, suggesting that apoptosis of T cells mediated through Fas/FasL may be a marginal immunosuppressive function by microglia (84). TAMs can express several chemokines such as CCL2, 5, 20 and 22 to enhance the recruitment of regulatory T cells (Tregs) (85–87). Tregs subsequently inhibit the activity of CD4^+^ and CD8^+^ effector T cells, natural killer (NK) cells, NKT cells, and antigen-presenting cells (APC) through a variety of mechanisms (85–87). It was recently described that TAMs overexpressed the enzymes indoleamine 2,3-dioxygenase 1 and tryptophan 2,3-dioxygenase 2 (IDO1/TDO2) to promote immunosuppression in GBM. L-Kynurenine (KYN) is a tryptophan-derived metabolite as a result of the enzymatic activity of IDO1/TDO2. KYN activates aryl hydrocarbon receptor (AHR) in TAMs to promote CCR2 expression, CD8^+^ T cell dysfunction and the generation of Tregs (88, 89).

## Tumor-associated microglia/macrophages as therapeutic targets in glioblastoma

### Depletion of TAMs

**Figure 4:**
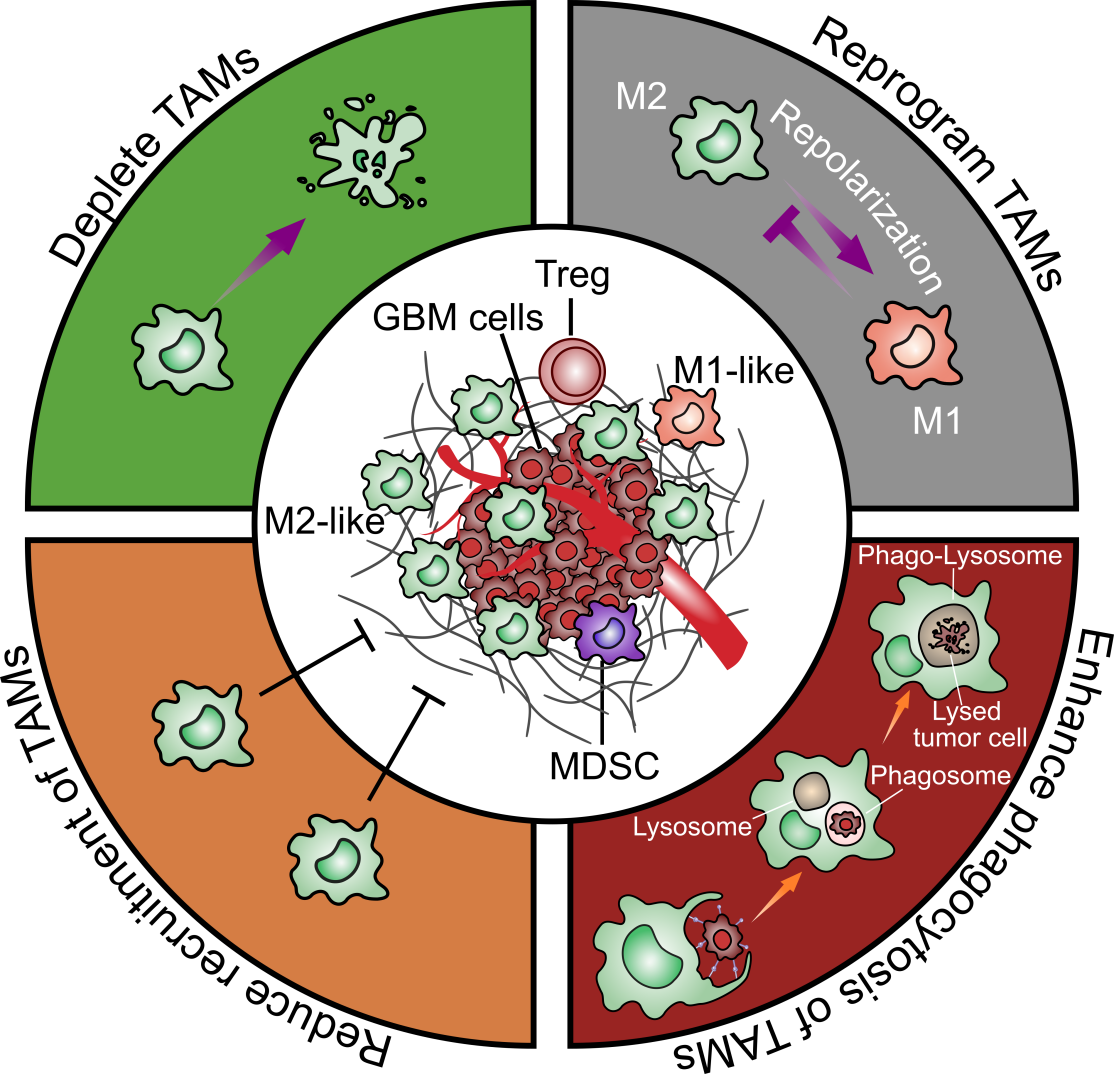
Strategies to target glioblastoma-associated microglia and macrophages. There are four general therapeutic strategies to target or utilize TAMs in GBM treatment including 1) directly depleting TAMs; 2) reprograming TAMs from an M2-like pro-tumoral phenotype to an M1-like anti-tumoral phenotype; 3) enhancing TAMs phagocytosis on tumor cells; 4) reducing TAMs recruitment to the tumor sites.

Based on the multiple pro-tumor functions of TAMs, they are considered as an important potential therapeutic target. Possible approaches such as depleting TAMs, repolarizing M2-like to M1-like TAMs, enhancing phagocytic activity of TAMs and reducing recruitment of TAMs will be discussed in the following (**Figure 4**).

Due to the high infiltration of TAMs within GBM and thereby driving tumor progression, several methods to deplete TAMs have been investigated. For example, in the CD11b-HSVTK (herpes simplex virus thymidine kinase) mouse model, infusion of ganciclovir performs microglia depletion *in vivo* (90). Using this model, vessel density and tumor volume were decreased in GL261-bearing mice (46, 64). Interestingly, selective depletion of resident microglia induced similar results compared to the reduction of the whole myeloid cell population, suggesting that resident microglia rather than peripheral macrophages are more crucial in promoting vascularization in GBM (64). On the contrary, macrophages can also slow the progression of malignant gliomas. TAMs depletion by ganciclovir contributed to a 33% tumor increase in the GBM syngeneic GL261 mouse model (35). Liposome-encapsulated clodronate, which can selectively deplete microglia, reduced the invasiveness of GBM in GL261 cultured brain slices. Inoculation of exogenous microglia can restore the invasiveness behavior (91). However, administration of liposome-encapsulated clodronate into brain parenchyma can also cause severe damage to other brain cells and blood vessel integrity (92). Selectively limiting peripheral macrophage infiltration *via* genetic Ccl2 depletion prolonged the survival of tumor-bearing mice (93). A silico studies based on real patients parameters have found that depletion of TAMs may be beneficial only for the patients who received the therapy in the early-stage GBM (94).

A major limitation of these studies is that the depletion of TAMs was achieved before the gliomagenesis. Thus, these findings lack translatability because of the totally different situations occurring in GBM patients. Given the high plasticity and heterogenous of TAMs in the GBM microenvironment, depleting the total TAMs pool may not be the optimal strategy.

### Repolarization of TAMs

In addition to depleting TAMs, it may be more efficient to repolarize M2-like to M1-like phenotype and acquire anti-tumorigenic functions.

CSF-1 secreted by glioma cells is essential for the differentiation and survival of TAMs and facilitates M2 polarization of TAMs (55, 56). Therefore, blocking the CSF-1 or its receptor CSF-1R is another potential therapeutic strategy for GBM (55, 95). In a mouse proneural GBM model, CSF-1R blockade significantly inhibited GBM tumor growth and prolonged survival. At the same time, expression of M2 markers decreased in TAMs (55). However, although CSF-1R significantly prolonged overall survival of GBM-bearing mice, tumors eventually recurred in more than 50% of mice.

In recurrent GBM, the phosphatidylinositol 3-kinase (PI3K) pathway activity, driven by macrophage-secreted insulin-like growth factor-1 (IGF-1) and tumor cell IGF-1 receptor (IGF-1R), was increased. To break the resistance state to CSF-1R inhibitors, blocking PI3K and IGF-1R is combined with CSF-1R inhibition, resulting in significantly suppressed tumor growth and prolonged overall survival (96). In addition, transcription factor STAT3 is another target to repolarize the TAMs to the M1-like phenotype. Inhibition of STAT3 in tumor TAMs can activate M1-like phenotype and reverse cytokine expression profile to anti-tumorigenic function, resulting in GBM growth delay (38, 97, 98).

The nanoparticles delivering mRNAs encoding interferon regulatory factor-5 (IRF-5) and its activating kinase IKKβ were infused into glioma-bearing mice. These nanoreagents reversed the pro-tumor state of TAMs, repolarized them to an M1-like phenotype and promoted tumor regression (99). Recently, a study showed that IL-33 is expressed in both human GBM specimens and murine models. A positive correlation between IL-33 expression and M2-like phenotype markers was observed. Inhibiting IL-33 expression dramatically suppressed GBM growth and prolonged survival (100).

### Enhancing phagocytosis of TAMs

Through phagocytosis, APCs are able to capture and eliminate tumor cells and present the tumor-derived antigens to prime T cells. However, GBM cells can overexpress the anti-phagocytosis molecule (don’t eat me signals) such as CD47 which binds to signal regulatory protein alpha (SIRPα), an inhibitory receptor expressed on TAMs to inhibit phagocytosis by TAMs (101–103). Thus, inhibiting the CD47-SIRPα anti-phagocytic axis may be a promising strategy to enhance tumor phagocytosis and activate the adaptive immune system.

Hu5F9-G4, a humanized anti-CD47 antibody, can block the anti-phagocytic CD47-SIRPα interaction. It enhanced tumor phagocytosis and reduced tumor burden in preclinical xenografts of various human malignancies, including GBM (103). In addition, in an immunocompetent mouse glioma model, blocking the CD47-SIRPα axis by anti-CD47 antibody significantly increased phagocytosis of glioma cells and GSCs by macrophages, consequently inhibiting tumor growth and prolonging survival (104). By using orthotopically xenografted, immunodeficient and syngeneic mouse models with genetically color-coded macrophages (Ccr2RFP) and microglia (Cx3cr1GFP), it was demonstrated that, in addition to macrophages, resident microglia within the TAMs pool are also effector cells of GBM cells phagocytosis in response to anti-CD47 blockade. Additionally, microglia induced a less inflammatory response compared to peripheral macrophages, making them a promising target for clinical applications (102). CD47 knockout increased tumor-associated extracellular matrix protein tenascin C (TNC) in U87 intracranial xenografts. Its overexpression in GBM cells is mediated by the Notch pathway. Inhibition of TNC reduced the phagocytosis of CD47-/- tumor cells in cocultures and enhanced the growth of CD47-/- xenografts *in vivo*. This suggests that TNC can be a potential target to enhance phagocytosis mediated by CD47 blockade in GBM (105).

However, CD47 blockade alone is inefficient in stimulating glioma cells phagocytosis by TAMs and has limited anti-tumor effects. Combining TMZ with CD47 blockade enhances the glioma cells phagocytosis and increases antigen cross-presentation, leading to more efficient T cells priming and anti-tumor immune responses *in vivo*. This combo treatment also activates immune checkpoint which can be turned off by sequential administrations of an anti-PD1 antibody (101).

### Reducing recruitment of TAMs

Due to the numerous chemoattractants existing in the TME of GBM, which can recruit TAMs to promote tumor progression, it is a promising therapy to reduce the recruitment of tumor-promoting TAMs.

The CX3CL1/CX3CR1 chemokine axis is crucial for the recruitment of TAMs. In response to activation of CX3CL1, CX3CR1-expressing TAMs can be recruited to promote GBM progression. In contrast, after blocking the CX3CL1/CX3CR1 system with neutralizing anti-CX3CL1 or CX3CR1 antibodies, recruitment of TAMs was significantly inhibited (106). Periostin secreted by GSCs can promote the recruitment of TAMs through the integrin α_v_β_3_ in human GBM. Inhibiting periostin markedly decreased TAMs recruitment, suppressed tumor growth and prolonged survival of mice bearing GSC-derived xenografts (107). The activation of stromal cell-derived factor-1 (SDF-1/CXCL12) and its receptor, CXCR4, is essential for TAMs recruitment (108–110). Using U87-MG intracranial xenografts, inhibitors of CXCR4 signaling such as peptide R and Plerixafor modulated the TAMs towards an anti-tumorigenic phenotype and inhibited the growth of glioma cells *in vitro* (111). In addition, CCL2 (MCP-1) produced by the GBM microenvironment recruits microglial cells to GBM and promotes their growth *in vivo* (112).

OPN, secreted by GBM cells and TAMs, is another chemokine for recruiting TAMs to GBM and has the potential to be exploited. A positive correlation has been observed between OPN expression levels and glioma grades as well as the infiltration of TAMs. OPN blockade significantly impaired the ability of GBM to recruit TAMs, enhanced T cells effector activity and prolonged survival in GL261 glioma-bearing mice (113). Since the high expression levels of OPN are observed in TME, OPN can also be exploited as a homing molecule to the GBM TME (114, 115). For example, OPN aptamer triggered CD3^+^ T cells activation within the TME and prolonged the survival in mice bearing intracerebral GL261 tumors (113).

Three noncytotoxic drugs (an antibiotic - minocycline, an antihypertensive drug - telmisartan, and a bisphosphonate - zoledronic acid) have an inhibitory function on the MCP-1 synthesis and impede TAMs recruitment induced by MCP-1, thereby delaying GBM growth. Since these three older drugs have already been approved for treating infection, hypertension, and osteoporosis, respectively, they have the advantages of better safety and lower cost. They will be tested in a pilot clinical trial in primary glioblastoma patients (116). CSF-1 secreted by glioma cells can also support the recruitment of TAMs (55). Thus, CSF-1R inhibition has been receiving increasing attention (95, 117). Blockade of CSF-1R signaling by using the PLX3397 (a CSF-1R inhibitor) in glioma-bearing mice decreased the recruitment of TAMs and reduced the GBM invasion (56). However, these promising results was failed to translate into clinical application (118). No efficacy was observed in a phase II clinical trial (NCT01349036) in which PLX3397 was orally administered in recurrent GBM patients (118).

### Clinical trials targeting TAMs in GBM

Based on the above preclinical studies, many clinical trials are being initiated by targeting TAMs for the treatment of GBM. Emactuzumab (RG7155), a therapeutic anti-CSF-1R antibody, has been combined with the programmed cell death-1 ligand (PD-L1)-blocking mAb atezolizumab in a phase I study (NCT02323191), in which a considerable ORR was particularly seen with a manageable safety profile. Another key target for CSF-1R blockage is the transcription factor STAT3. In a phase I clinical trial (NCT01904123), the side effects and best dose of STAT3 inhibitor WP1066 were evaluated in patients with recurrent malignant glioma. Macrophage migration inhibitory factor (MIF) is overexpressed in many tumors, including GBM. MIF induces angiogenesis, promotes cell cycle progression, and inhibits apoptosis (119). An ongoing phase I/II clinical trial (NCT03782415) is evaluating Ibudilast (a MIF inhibitor) and TMZ combo treatment in newly diagnosed and recurrent GBM. As mentioned before, the SDF-1/CXCL12 axis is essential for TAMs recruitment. Therefore, a pilot phase I/II trial (NCT01977677) studied the side effects and best dose of Plerixafor (a CXCR4 inhibitor) after radiation therapy plus TMZ to see how well it works in treating patients with newly diagnosed high-grade glioma. **Table 1** summarizes the clinical trials of monotherapies or combinatorial approaches targeting TAMs in GBM.

**Table 1:** Clinical trials targeting TAMs in GBM.

Trial Name (Identifier)	Target/Function	Drug Name	Additional Treatment	Phase of Trial	Tumor Type
NCT02323191	CSF-1R inhibitor	RG7155 (Emactuzumab)	Atezolizumab (anti-PD-L1)	I	GBM
NCT01790503	CSF-1R inhibitor	PLX3397 (Pexidartinib)	RT + TMZ	I/II	GBM
NCT02526017	CSF-1R inhibitor	Cabiralizumab	Nivolumab (anti-PD-1)	I	GBM
NCT01977677	CXCR4 antagonist	Plerixafor (AMD3100)	RT+TMZ	I/II	GBM
NCT02765165	CXCR4 inhibitor	USL311	Lomustine	II	rGBM
NCT01349036	CSF-1R inhibitor	PLX3397 (Pexidartinib)	–	II	rGBM
NCT03341806	PD-L1 inhibitor	Avelumab	MRI-guided LITT therapy	I	rGBM
NCT01339039	CXCR4 antagonist	Plerixafor (AMD3100)	Bevacizumab	I	rGBM
NCT01904123	STAT3 inhibitor	WP1066	–	I	rGBM
NCT03382977	GM-CSF	VBI-1901	–	I/II	rGBM
NCT02829723	CSF-1R inhibitor	BLZ945	PDR001 (anti-PD-1)	I/II	GBM/rGBM
NCT03782415	MIF inhibitor	Ibudilast	TMZ	I/II	GBM/rGBM

TAMs: tumor-associated microglia/macrophages; CSF-1R: colony-stimulating factor 1 receptor; PD-L1: programmed cell death-Ligand 1; CXCR4: CXC motif chemokine receptor 4; STAT3: signal transducer and activator of transcription 3; GM-CSF: granulocyte-macrophage colony-stimulating factor; MIF: Macrophage migration inhibitory factor; RT: radiotherapy; TMZ: temozolomide; MRI: magnetic resonance imaging; LITT: laser interstitial thermal therapy; GBM: glioblastoma; rGBM: relapsed/recurrent glioblastoma.

## Conclusions and Perspectives

Decades of investigations have been conducted to focus on the tumor cells themselves while less attention has been paid to the components of TME. With the accumulation of knowledge about the TME, TAMs have emerged as exciting targets for therapeutic intervention. Although many investigations have contributed substantially to our understanding of the origin, polarization and functional heterogeneity of TAMs, it is still unclear upon the complex interaction and dynamics between GBM and TAMs.

Many researches have demonstrated that macrophages and microglia within the TAMs pool in GBM show distinct morphological and transcriptional changes. In addition, they both have high plasticity and can be modulated by the different TME. Thus, it is crucial to differentiate these two cell populations and understand their different roles in GBM tumorigenesis and progression. In order to successfully target the immunosuppressive M2-like TAMs population for GBM treatment, we also need to fully understand the complex interplay between TAMs and other immune cells within the TME. Furthermore, a better understanding of the bridge-like role of TAMs between innate immune and adaptive immune system is indispensable for exploiting TAMs to activate an anti-tumor immune response.

Targeting TAMs has proven to be a promising strategy in preclinical trials. This strategy could not only suppress the germination of GBM “seeds” but also wreak the “fertile soil” of GBM, further destroying the immunosuppressive microenvironment, thus inhibiting the tumor growth. With the development of other immunotherapeutic strategies, novel synergistic combinations of TAMs-targeting therapeutics and other immunotherapies may ultimately support the eradication of GBM. Currently, extensive exploration and development of TAMs-targeting agents have been ongoing, among which some have been utilized in clinical trials. Hence, there is no doubt that TAMs-targeting strategies will benefit more GBM patients in the future.

## Author Contributions

All authors contributed to gathering of data, writing, editing, and revising of the manuscript.

## Funding

This work was supported by the National Natural Science Foundation of China (81772693 and 82073404) and the Youth fund of National Natural Science Fund of China (82102898).

## Acknowledgements

We thank our colleagues for stimulating discussions.

## Conflict of Interest

The authors declare that the research was conducted in the absence of any commercial or financial relationships that could be construed as a potential conflict of interest.

## Publisher’s Note

All claims expressed in this article are solely those of the authors and do not necessarily represent those of their affiliated organizations, or those of the publisher, the editors and the reviewers. Any product that may be evaluated in this article, or claim that may be made by its manufacturer, is not guaranteed or endorsed by the publisher.
